# Erythrocyte sedimentation rate in heartworm naturally infected dogs “with or without” *Leishmania infantum* seropositivity: an observational prospective study

**DOI:** 10.3389/fvets.2024.1371690

**Published:** 2024-03-15

**Authors:** Maria Alfonsa Cavalera, Oana Gusatoaia, Annamaria Uva, Floriana Gernone, Viviana Domenica Tarallo, Rossella Donghia, Marco Silvestrino, Andrea Zatelli

**Affiliations:** ^1^Department of Veterinary Medicine, University of Bari, Bari, Italy; ^2^National Institute of Gastroenterology - IRCCS “Saverio de Bellis”, Bari, Italy

**Keywords:** ESR, APP, inflammatory markers, *Dirofilaria immitis*, canine leishmaniosis, coinfections

## Abstract

Canine heartworm disease by *Dirofilaria immitis* and canine leishmaniosis by *Leishmania infantum* (CanL) are both vector-borne diseases with frequently overlapping endemicity and able to trigger the acute phase response, being characterized by variations in acute phase proteins (APP). Recently, erythrocyte sedimentation rate (ESR), an indicator of inflammation, has gained attention in veterinary medicine, proving useful in several conditions that include CanL active forms in dogs. This study aims to evaluate ESR in heartworm-infected dogs, compare levels with heartworm-infected and *L. infantum* seropositive dogs as well as clinically healthy dogs, and assess correlations with other laboratory parameters. From October 2022 to January 2023, a prospective observational study was conducted enrolling heartworm-infected (*Dirofilaria* group) and heartworm-infected *L. infantum* seropositive (*Dirofilaria*/*Leishmania* group) animals subgrouped according to the CanL clinical form (*Dirofilaria*/*Leishmania* active and non-active groups). A group of clinically healthy dogs (control group) was also included. For each dog enrolled physical examination and laboratory tests (complete blood count, biochemical panel including APP, serum protein electrophoresis) were performed. *Dirofilaria* and *Dirofilaria*/*Leishmania* groups presented a significantly higher ESR level compared to healthy dogs. *Dirofilaria*/*Leishmania* active group had the highest ESR level among the groups considered. *Dirofilaria*/*Leishmani*a non-active group had an ESR similar to the *Dirofilaria* group, but significantly higher and lower compared to the control and the *Dirofilaria*/*Leishmania* active group, respectively. A significant positive correlation between ESR and C-Reactive Protein has been found in all groups except for the *Dirofilaria*/*Leishmania* non-active group. In *Dirofilaria*/*Leishmania* active group a strong positive correlation between ESR and gamma globulins percentage as well as a strong negative correlation between ESR and albumin, albumin/globulins ratio were found. Overall, the ESR was confirmed to be an inflammation marker as well as a helpful disease index, being notably increased in heartworm-infected dogs affected by an active form of CanL.

## 1 Introduction

Canine heartworm disease (HWD) is a potentially life-threatening mosquito-borne disease caused by the nematode *Dirofilaria immitis*, affecting both wild and domestic dogs with worldwide distribution and global significance (1, 2). Heartworms reside in the pulmonary arteries of the canine definitive host, causing proliferative endarteritis which can progress to chronic vascular remodeling, pulmonary hypertension, and right-sided congestive heart failure (1, 3). The pathophysiology of the HWD damage is based on the host’s response to the parasite [i.e., adults and microfilariae (mfs)] and the bacterial endosymbiont *Wolbachia pipientis*, which resides in all biological stages of *D. immitis*, triggering the release of proinflammatory and chemotactic cytokines and leading to cellular infiltration and amplification of the inflammatory response (4, 5). The host’s initial immune interaction against the parasite falls under the *umbrella* of the innate immune response which includes the development of an acute phase response (APR) (6, 7), being a nonspecific and complex reaction that happens shortly after any tissue injury (e.g., trauma, infection, or inflammation) trying to restore the homeostasis and eliminate the underlying cause of the disturbance (8, 9). In this context, an APR characterized by a variation in the acute phase proteins (APP) concentrations such as an increase in C-reactive protein (CRP) not accompanied by increases in haptoglobin as positive APP, and a decrease in albumin and paraoxonase-1 (PON-1) as negative APP have been described in dogs infected by *D. immitis* (6, 7, 10). Moreover, a CRP increase according to the degrees of the disease and a correlation between positive APP such as CRP and the severity of pulmonary arteriola damage and pulmonary hypertension have been also reported (11–13), indicating inflammatory processes could contribute to the progression of the disease. Therefore, though more research is needed, inflammation biomarkers such as APP seem promising in assessing the status of the heartworm-infected animals, diagnosing pathological aspects of the HWD and/or helping in the establishment of an accurate prognosis (7).

In this scenario, the ESR measurement is a hematology test commonly performed in human medicine to measure the speed at which red blood cells (RBC) settle into a tube of anticoagulated blood in a specific unit of time, most commonly an hour, to highlight the occurrence and extent of inflammation (14, 15). This determination has recently returned to the spotlight in veterinary medicine, being identified as a useful and timely inflammatory biomarker in cases of canine rheumatoid arthritis, osteoarthritis, babesiosis, ehrlichiosis, and feline chronic kidney disease (16–20).

In addition, ESR proved to be a sensitive biomarker for the diagnosis of active forms of canine leishmaniosis by *Leishmania infantum* (CanL) (21), a sand fly-borne disease that recognizes an APR as part of innate immunity. Similarly to HWD, the inflammatory response that characterizes CanL in active form consists of increases in positive APP, such as CRP, and decreases in negative APP such as albumin, paraoxonase 1, or apolipoprotein 1. Moreover, the endemicity of CanL frequently overlaps with that of HWD (22), leading to the high possibility of co-infections in endemic areas (23).

Hence, this study aims: (i) to evaluate the ESR in dogs naturally infected by *D. immitis*; (ii) to compare the ESR level in heartworm-infected dogs with a population of heartworm-infected and *L. infantum* seropositive dogs as well as with a group of clinically healthy dogs; (iii) to assess the existence of a correlation between ESR and other laboratory parameters (e.g., APP) in heartworm naturally infected dogs.

## 2 Materials and methods

### 2.1 Study area and enrolment criteria

This prospective observational study was conducted in a rescue shelter located in southern Italy (40.419326^°^ N, 18.165582^°^ E, Apulian region, Lecce), an endemic area for *D. immitis* and *L. infantum* (22). From October 2022 to January 2023, heartworm-infected (i.e., “*Dirofilaria* group”) and heartworm-infected *L. infantum* seropositive (i.e., “*Dirofilaria*/*Leishmania* group”) dogs were enrolled in the study. Dogs were considered heartworm-infected if positive on the modified Knott’s test for *D. immitis* mfs or to the enzyme-linked immunosorbent assay (ELISA) for *D. immitis* Ag. Moreover, in the *Dirofilaria*/*Leishmania* group, heartworm-infected dogs were considered *L. infantum* seropositive if they tested positive on an indirect immunofluorescence antibody test (IFAT) for *L. infantum* antibodies.

Based on physical examination, clinical score, and laboratory results, being performed as described in the following, a cohort of dogs considered clinically healthy has been also included in the “control group.” These dogs tested seronegative for *L. infantum*, *Anaplasma phagocytophilum* and *Ehrlichia canis* by IFAT, and negative for *D. immitis* by Knott’s test and ELISA. More, dogs belonging to the *Dirofilaria*/*Leishmania* group were further subgrouped according to the CanL clinical form as affected by a “non-active” (i.e., absence of clinical signs and laboratory alterations compatible with CanL active form; *Dirofilaria*/*Leishmania* non-active group) or “active” (i.e., presence of clinical signs and/or laboratory alterations compatible with CanL; *Dirofilaria*/*Leishmania* active group) form of CanL (24, 25).

Dogs were excluded if suspected or known to be: (i) affected by diseases or treated with drugs able to influence the immune response and the inflammatory markers (e.g., neoplastic, auto-immune and heart diseases, diabetes mellitus and insipidus, hypo- and hyperadrenocorticism or hypothyroidism, anti-inflammatory, and/or immunosuppressive drugs); (ii) affected by diseases able to influence the ESR according to the current evidence (i.e., canine rheumatoid arthritis, osteoarthritis, and babesiosis) (16–19); (iii) affected by other vector-borne pathogens such as *A. phagocytophilum* and *E. canis*, or infected by other filarial nematodes namely *Dirofilaria repens* and *Acanthocheilonema reconditum*. Any aggressive, debilitated, or severely sick animals as well as pregnant females were excluded from the study.

### 2.2 Procedures

For each animal included, signalment data (i.e., age, sex, and breed), clinical history, and, if available, previous laboratory analyses have been recorded in individual files. A complete physical examination including body condition score (BCS) and muscle condition score (MSC) evaluation based on a 9 and 4-point scale, respectively, was performed (26, 27). Clinical signs compatible with HWD according to the major guidelines (28–30) were reported in the patient’s medical record. Heartworm-infected dogs were considered symptomatic if the presence of one or more clinical signs related to HWD (i.e., dyspnea, cough, exercise intolerance, weakness, loss of weight, and syncope) were observed as well as signs related to right-sided congestive heart failure (i.e., ascites, jugular venous distension, and hepatomegaly) (28–30). Additionally, a CanL-dedicated clinical score following a previously validated assessment scale ranging from 0 (i.e., absence of clinical signs) to 19 (31) was assigned.

Blood samples were collected from either the cephalic or jugular veins and placed in a K3 EDTA tube (2 mL) to undergo ESR evaluation and complete blood count (CBC), while a blood aliquot (5 mL) was placed in plain tubes to obtain serum after centrifugation (15 min at 1,500 × g) and perform biochemical panel including APP (i.e., CRP and serum ferritin) and capillary zone electrophoresis. Furthermore, serum samples were tested for anti-*A. phagocytophilum* and anti-*E. canis* antibodies by IFAT and excluded in case of positivity.

### 2.3 Laboratory analyses

The modified Knott’s test used to detect circulating mfs in whole-blood samples was performed according to Genchi et al. (32). In the case of positivity, after the mfs identification based on morphological keys (33), a duplex real-time quantitative PCR (qPCR) was used to differentiate *Dirofilaria* species (34). In case of negative results with the modified Knott’s test, serum samples were analyzed for the presence of *D. immitis*–specific antigens by using an ELISA on microplate (Filarcheck, Agrolabo, Scarmagno, Italy).

The IFAT for *L. infantum* antibodies was performed according to Iatta et al. (35). Samples were considered positive when they produced a clear cytoplasmic and membrane fluorescence of promastigotes from a cut-off dilution of 1:80.

To evaluate the ESR of the dogs, a point-of-care device (MINIPET, DIESSE, Diagnostica Senese S.p.A., Siena, Italy) was used according to Militello et al. (36). The reference interval of ESR was established as 1–8 mm/h in 14 min (37).

The results of CBC (Siemens, ADVIA 2120, Erlangen, Germany), serum biochemical analysis (Beckman Coulter, Clinical Chemistry Analyzer AU680, Indianapolis, United States), and serum protein electrophoresis (SEBIA Italia S.r.l., Capillarys 2 Flex Piercing, Florence, Italy) were obtained by the same methods in all tested samples. Microscopic blood smear examination was also performed for all samples.

### 2.4 Statistical analysis

The normality of result distribution was assessed employing the Kolmogorov–Smirnov test. Descriptive statistics were used, presenting results as mean and standard deviation (*M* ± *SD*) or median and interquartile range for normally or non-normally distributed data, respectively. Categorical variables were expressed as frequencies and percentages (%). To analyze associations between independent groups (i.e., *Dirofilaria* group, *Dirofilaria/Leishmania* group, *Dirofilaria/Leishmania* non-active group, *Dirofilaria/Leishmania* active group, and healthy group), the Wilcoxon Rank Mann–Whitney test was utilized for continuous variables, and Fisher’s exact test for categorical variables. The Kruskal–Wallis equality rank test was employed for comparisons involving more than two independent groups, and Dunn’s test facilitated multiple pairwise comparisons. The Spearman rank correlation coefficient assessed the strength and direction of associations between examined variables. The correlation coefficient (ρ) was categorized as weak (*r*s = 0–0.3), moderate (*r*s = 0.3–0.6), strong (*r*s = 0.6–0.9), or very strong (*r*s = 0.9–1). Results were presented as rho correlation and associated *p*-value in brackets. The null hypothesis of no association was tested at a significance level of 0.05 (two-tailed). All statistical analyses were conducted using StataCorp. 2023. Stata Statistical Software: Release 18. College Station, TX: StataCorp LLC.

## 3 Results

A total of 98 shelter dogs were screened for enrollment. Twenty-nine dogs were excluded being heartworm-negative and not clinically healthy (*n* = 27), or positive to anti-*Anaplasma* antibodies (*n* = 2). Sixty-nine [*n* = 36 males and *n* = 33 females; 7.0 (1.3–9.0) years] out of 98 dogs met the inclusion criteria and were consequently enrolled in the study, including 22 animals in the control group, 18 in the *Dirofilaria* group, and 29 in the *Dirofilaria*/*Leishmania* group. Of the last group, 21 out of 29 dogs were affected by a CanL non-active form (i.e., *Dirofilaria*/*Leishmania* non-active group) whereas the remaining 8 were diagnosed with an active form of CanL (i.e., *Dirofilaria*/*Leishmania* active group).

**Figure 1:**
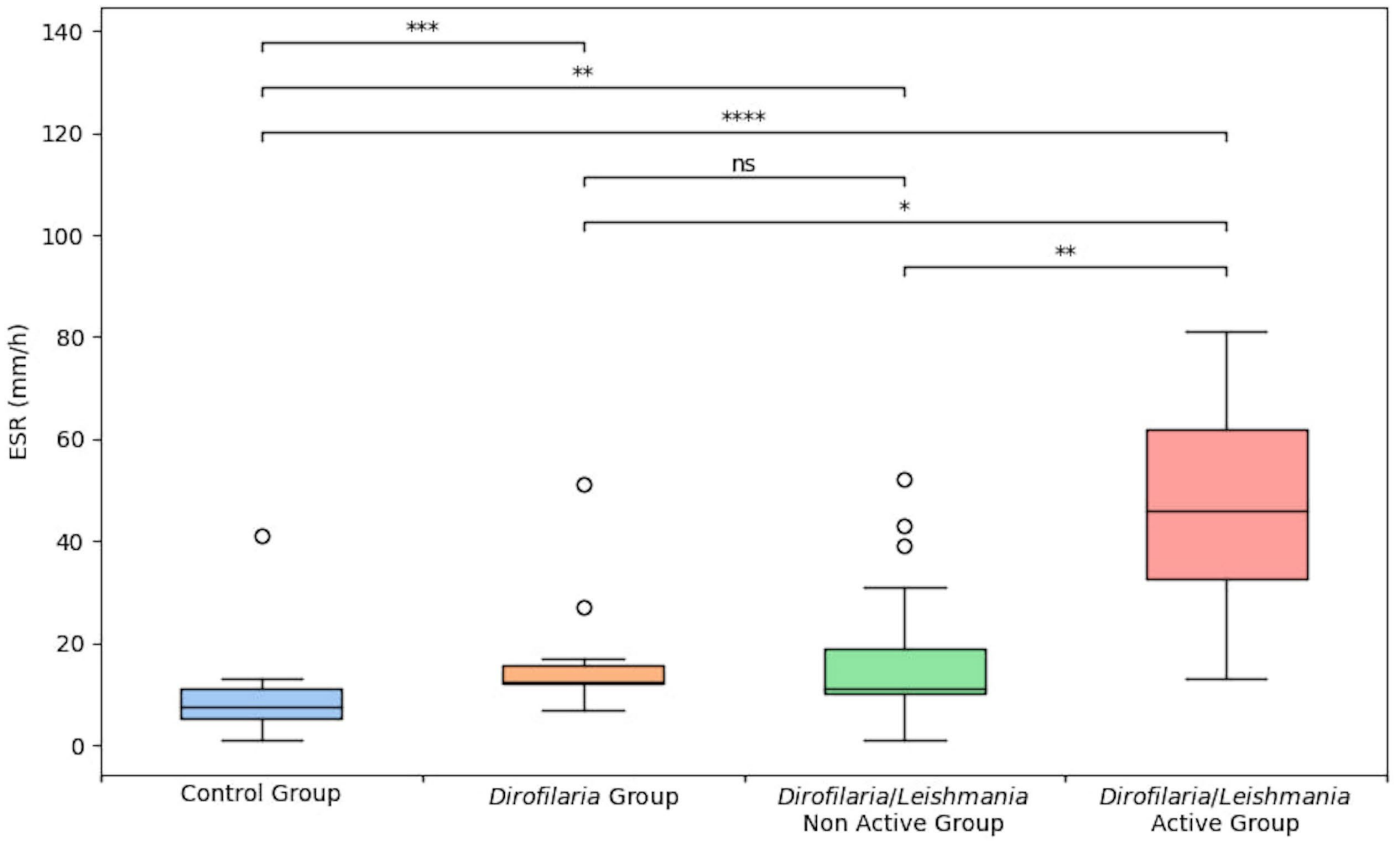
Erythrocyte sedimentation rate level in healthy dogs (i.e., control group), heartworm-infected dogs (i.e., *Dirofilaria* group), heartworm-infected *L. infantum* seropositive dogs affected by a non-active form of leishmaniosis (i.e., *Dirofilaria*/*Leishmania* non-active group), and heartworm-infected *L. infantum* seropositive dogs affected by an active form of leishmaniosis (i.e., *Dirofilaria*/*Leishmania* active group). The box plots show the median (line within the box), 25th and 75th percentiles (box) and minimum and maximum values (whiskers). Asterisks indicated significant differences between groups: ^*^p < 0.05, ^**^P < 0.01, ^***^p < 0.001, ^****^p < 0.0001.

Erythrocyte sedimentation rate level and the proportion of animals with ESR values higher than 8 mm/h in *Dirofilaria*, *Dirofilaria*/*Leishmania*, and control groups were statistically compared and reported in Table 1. The same comparison including the subclasses of the *Dirofilaria*/*Leishmania* group is detailed in Table 2 and Figure 1.

**Table 1:** Comparison of the erythrocyte sedimentation rate (ESR) level and the proportion of dogs with altered ESR level in healthy dogs (i.e., control group), heartworm-infected dogs (i.e., *Dirofilaria* group), and heartworm-infected *L. infantum* seropositive dogs (i.e., *Dirofilaria*/*Leishmania* group).

Parameters^*^	Control group (*n* = 22) (*a*)	*Dirofilaria* group (*n* = 18) (*b*)	*Dirofilaria*/*Leishmania* group (*n* = 29) (*c*)	*p* ^^^	Comparisons^†^		
					(*b*) vs. (*a*)	(*c*) vs. (*a*)	(*c*) vs. (*b*)
ESR	7.5 (5–11)	12.5 (12–16)	13 (11–41)	**0.0001**	**0.0002**	**<0.0001**	0.43
ESR > 8.0 mm/h	10 (45.4)	16 (88.9)	26 (89.6)	**0.001** ^ѱ^	**0.004** ^¥^	**0.001** ^¥^	0.99^¥^

*As median and interquartile range (IQR) for continuous variables, and as frequency and percentage (%) for categorical variables. ^Kruskal–Wallis rank test; ^ѱ^Fisher Exact test; ^†^Dunn’s test; ^¥^Proportion test. The bold values indicate the statistically significant results.

**Table 2:** Comparison of the erythrocyte sedimentation rate (ESR) level and the proportions of dogs with altered ESR level in healthy dogs (i.e., control group), heartworm-infected dogs (i.e., *Dirofilaria* group), heartworm-infected *L. infantum* seropositive dogs affected by a non-active form of leishmaniosis (i.e., *Dirofilaria*/*Leishmania* non-active group), and heartworm-infected *L. infantum* seropositive dogs affected by an active form of leishmaniosis (i.e., *Dirofilaria*/*Leishmania* active group).

Parameters*	Control group (*n* = 22) (*a*)	*Dirofilaria* group (*n* = 18) (*b*)	*Dirofilaria*/*Leishmania* non-active group (*n* = 21) (*c*)	*Dirofilaria*/*Leishmania* active group (*n* = *8*) (*d*)	*p^*	Comparisons^†^					
						(*b*) vs. (*a*)	(*c*) vs. (*a*)	(*d*) vs. (*a*)	(*c*) vs. (*b*)	(*d*) vs. (*b*)	(*d*) vs. (*c*)
ESR	7.5 (5–11)	12.5 (12–16)	11 (10–16)	46 (27–62)	**0.0001**	**0.0002**	**0.004**	**<0.0001**	0.18	**0.01**	**0.001**
ESR > 8.0 mm/h	10 (45.4)	16 (88.9)	18 (85.7)	8 (100.0)	**0.002** ^ѱ^	**0.004** ^¥^	**0.006** ^¥^	**0.007** ^¥^	0.77^¥^	0.33^¥^	0.26^¥^

*As median and interquartile range (IQR) for continuous variables, and as frequency and percentage (%) for categorical variables. ^Kruskal–Wallis rank test; ^ѱ^Fisher Exact test; ^†^Dunn’s test; ^¥^Proportion test. The bold values indicate the statistically significant results.

*Dirofilaria* and *Dirofilaria*/*Leishmania* groups presented a significantly higher ESR level (*p* = 0.0002 and *p* < 0.0001, respectively) compared to healthy dogs (Table 1). *Dirofilaria*/*Leishmania* group did not show a significant difference in ESR median value than the *Dirofilaria* group (Table 1). However, when the CanL subclasses were considered, heartworm-infected dogs affected by a CanL active form (i.e., *Dirofilaria*/*Leishmania* active group) had a significantly higher ESR level compared to all the other groups examined (Table 2). Differently, heartworm-infected dogs affected by a CanL non-active form (i.e., *Dirofilaria*/*Leishmani*a non-active group) revealed an ESR similar to the *Dirofilaria* group, but significantly higher and lower compared to the control and the *Dirofilaria*/*Leishmania* active group, respectively (Table 2).

The percentage of animals with an ESR level >8 mm/h was significantly higher in *Dirofilaria* and *Dirofilaria*/*Leishmania* groups including the CanL subclasses than in healthy dogs (Table 1), while no other differences among groups have been found (Table 1).

Correlations between ESR and laboratory parameters including inflammatory markers are shown in Table 3. A significant from moderate to strong positive correlation between ESR and CRP has been found in all groups except for the *Dirofilaria*/*Leishmania* non-active group. Furthermore, in *Dirofilaria*/*Leishmania* group a moderate positive correlation between ESR and globulins (*p* = 0.008) and beta globulins percentage (*p* = 0.04) as well as a moderate negative correlation between ESR and HCT (*p* = 0.03), albumin (*p* = 0.04), albumin percentage (*p* = 0.01), and albumin/globulins ratio (*p* = 0.01) were detected (Table 3). In *Dirofilaria*/*Leishmania* active group a strong positive correlation between ESR and gamma globulins percentage (*p* = 0.02) as well as a strong negative correlation between ESR and albumin (*p* = 0.01), albumin percentage (*p* = 0.01), albumin/globulins ratio (*p* = 0.04) were found (Table 3). In *Dirofilaria*/*Leishmania* non-active group, no correlations between ESR and the laboratory parameters considered were present (Table 3).

**Table 3:** Correlation matrix between ESR and other parameters recorded.

Parameters	*Dirofilaria* group ρ (*p*-value)	*Dirofilaria*/*Leishmania* group ρ (*p*-value)	*Dirofilaria*/*Leishmania* non-active group ρ (*p*-value)	*Dirofilaria*/*Leishmania* active group ρ (*p*-value)
HCT	−0.23 (0.35)	−0.41 (**0.03**)	−0.37 (0.10)	−0.43 (0.28)
Hemoglobin	−0.20 (0.43)	−0.34 (0.07)	−0.12 (0.59)	−0.43 (0.28)
RBC	−0.22 (0.38)	−0.29 (0.13)	−0.15 (0.50)	−0.23 (0.58)
CRP	0.61 (**0.009**)	0.39 (**0.04**)	0.16 (0.49)	0.73 (**0.04**)
HPT	0.30 (0.22)	−0.007 (0.97)	0.13 (0.58)	−0.31 (0.45)
Ferritin	−0.28 (0.25)	−0.01 (0.95)	−0.04 (0.85)	−0.18 (0.66)
Albumin	−0.25 (0.32)	−0.40 (**0.04**)	0.03 (0.90)	−0.86 (**0.01**)
Globulins	0.46 (0.06)	0.50 (**0.008**)	0.18 (0.44)	0.29 (0.48)
Albumin/globulins ratio	−0.38 (0.12)	−0.47 (**0.01**)	−0.08 (0.74)	−0.74 (**0.04**)
Total proteins	0.28 (0.25)	0.29 (0.14)	0.09 (0.71)	−0.02 (0.95)
Albumin %	−0.28 (0.25)	−0.48 (**0.01**)	0.07 (0.75)	−0.73 (**0.04**)
Alpha-2 globulins %	−0.05 (0.85)	0.04 (0.85)	0.02 (0.92)	0.42 (0.29)
Beta globulins	−0.12 (0.64)	0.38 (**0.04**)	0.07 (0.75)	−0.26 (0.52)
Beta-2 globulins %	0.15 (0.53)	0.18 (0.37)	0.02 (0.92)	−0.37 (0.35)
Beta-3 globulins %	−0.08 (0.75)	0.22 (0.26)	−0.03 (0.90)	−0.17 (0.68)
Gamma globulins %	0.32 (0.19)	0.21 (0.28)	−0.18 (0.44)	0.80 (**0.02**)

ρ, Spearman’s Rho coefficient and relative *p*-value. The bold values indicate the statistically significant results. CRP, C-reactive protein; HCT, hematocrit; HPT, haptoglobin; RBC, red blood cells.

Values of the main inflammatory markers evaluated (i.e., CRP, HPT, ferritin, albumin, and ESR) in *Dirofilaria* group, and *Dirofilaria*/*Leishmania* group including the two subclasses were statistically compared and reported in Table 4. Among positive APP, CRP level was significantly increased in the *Dirofilaria*/*Leishmania* active group where also the number of the dogs with an increased value was statistically higher compared to the other two groups (Table 4). In the *Dirofilaria*/*Leishmania* active group, the number of animals with increased ferritin was also significantly higher than in the *Dirofilaria*/*Leishmania* non-active group (Table 4). No statistically significant difference was found between the groups for haptoglobin (Table 4). Albumin was significantly decreased in the *Dirofilaria*/*Leishmania* active group in which 87.5% of the animals had a significantly reduced value (Table 4).

**Table 4:** Comparison of the inflammatory markers and the number (percentage) of dogs with altered parameter levels in healthy dogs (i.e., healthy group), and *D. immitis* infected dogs (i.e., *Dirofilaria* group) as well as in heartworm-infected *L. infantum* seropositive dogs subclassified as affected by a non-active (i.e., *Dirofilaria*/*Leishmania* non-active group) and active (i.e., *Dirofilaria*/*Leishmania* active group) form of leishmaniosis according to the clinical form.

Parameters (RI)*	*Dirofilaria* group (*n* = 18) (*a*)	*Dirofilaria*/*Leishmania* non-active group (*n* = 21) (*b*)	*Dirofilaria*/*Leishmania* active group (*n* = 8) (*c*)	*p^^^*	Comparisons^†^		
					(*b*) vs. (*a*)	(*c*) vs. (*a*)	(*c*) vs. (*b*)
CRP (0.0–1.0 mg/dL)	0.81 (0.51–1.35)	0.56 (0.37–1.37)	1.53 (1.39–3.10)	0.06	0.17	0.06	**0.01**
CRP > 1.0 mg/dL	7 (38.89%)	6 (30.00%)	7 (87.50%)	**0.02** ^ѱ^	0.56^¥^	**0.02** ^¥^	**0.005** ^¥^
HPT (5.0–100.0 mg/dL)	4.00 (3.00–6.00)	10 (3.00–52.45)	5.55 (2.75–47.00)	0.48	0.12	0.24	0.41
HPT > 100 mg/dL	1 (5.56%)	2 (10.00%)	1 (12.50%)	0.82^ѱ^	0.61^¥^	0.54^¥^	0.84^¥^
HPT < 5.0 mg/dL	10 (55.56)	7 (33.33)	2 (25.00)	0.25^ѱ^	0.16^¥^	0.15^¥^	0.66^¥^
Ferritin (80–270 ng/mL)	148 (118.00–211.00)	151 (117.00–216.00)	201 (120.00–291.50)	0.66	0.43	0.22	0.18
Ferritin >270 ng/mL	2 (11.11%)	1 (5.00%)	3 (37.50%)	0.10^ѱ^	0.48^¥^	0.11^¥^	**0.02** ^¥^
Albumin (2.8–3.7 g/dL)	2.50 (2.40–2.90)	2.80 (2.60–3.05)	2.40 (2.20–2.50)	**0.02**	**0.04**	0.09	**0.004**
Albumin <2.8 g/dL	13 (72.22%)	9 (45.00%)	7 (87.50%)	0.08^ѱ^	0.09^¥^	0.39^¥^	**0.04** ^¥^
ESR (0.0–8.0 mm/h)	12.50 (12.00–16.00)	11.00 (10.00–16.00)	46.00 (27.00–62.00)	**0.002**	0.20	**0.002**	**0.0002**
ESR >8.0 mm/h	16 (88.89%)	18 (85.71%)	8 (100.00%)	0.83^ѱ^	0.77^¥^	0.33^¥^	0.26^¥^

*As median and interquartile range (IQR) for continuous variables, and as frequency and percentage (%) for categorical variables. ^Kruskal–Wallis rank test; ^ѱ^Fisher Exact Test; ^†^Dunn’s test; ^¥^Proportion test. CRP, C-reactive protein; HPT, haptoglobin; ESR, erythrocyte sedimentation rate. The bold values indicate the statistically significant results.

From a clinical point of view, dogs presented a good state of health with BCS and MCS mean values of 2.7 and 1.3, respectively. Moreover, enrolled dogs were considered asymptomatic for what concerns HWD, while animals in the *Dirofilaria*/*Leishmania* group showed a mean CanL clinical score of 1.0 (1.0 in the *Dirofilaria*/*Leishmania* non-active group and 1.2 in the *Dirofilaria*/*Leishmania* active group).

## 4 Discussion

In the present study, the ESR level has been evaluated in dogs naturally infected by *D. immitis* and compared to the values obtained from a group of healthy animals and a population of heartworm-infected and *L. infantum* seropositive dogs subclassified according to the CanL clinical form. Furthermore, the potential correlations between ESR and other inflammatory markers usually implied in clinical practice were also studied.

In the *Dirofilaria* group, the ESR median value (i.e., 12.5 mm/h *ca.*) was significantly higher compared to the healthy group (i.e., 7.5 mm/h *ca.*), confirming the existence of an APR in naturally heartworm-infected dogs which is known to be characterized by an increase in APP circulating in the blood, such as CRP. Indeed, in this group of heartworm-infected dogs, a positive correlation between ESR and CRP was found. However, while the CRP was increased in 38.9% of the dogs in the *Dirofilaria* group, the ESR level was higher in almost 89% of the cases.

When compared to the *Dirofilaria/Leishmania* group (ESR = 13 mm/h *ca.*), heartworm-infected dogs presented a slightly lower and not statistically different ESR value. However, this comparison yields markedly different results when considering the subgroups based on the clinical form of CanL within the *Dirofilaria/Leishmania* group. Indeed, while dogs in the *Dirofilaria* group exhibited an ESR value that was not statistically dissimilar from that of the heartworm-infected dogs affected by a CanL non-active form (i.e., 11 mm/h *ca.*), both these groups of animals showed significantly lower ESR levels compared to heartworm-infected dogs affected by a CanL active form (i.e., 46 mm/h *ca.*). Likely, these results mirror the clinical condition of the animals enrolled in this study. Considering that heartworm-infected dogs were asymptomatic for what concerns HWD, it has already been reported that the increase in APP such as CRP is more notable according to the severity of the disease, though the inflammatory response is significant from the early stages of the infection probably due to the endosymbiont *Wolbachia* spp. and the vascular damage (7, 11, 13). Similarly, dogs included in the *Dirofilaria*/*Leishmania* non-active group were asymptomatic for HWD and showed a low CanL clinical score.

Heartworm-infected dogs affected by a CanL active form showed the highest ESR median value (i.e., 46 mm/h *ca.*) among the groups considered in the present study. This finding is not surprising given that recent evidence demonstrated that dogs with a CanL active form had a higher ESR level compared to *L. infantum* exposed and healthy dogs (21). Additionally, the majority of these dogs in active form (92%) presented an increased ESR (21), as also herein reported in the *Dirofilaria/Leishmania* active group (100%). While direct comparison of ESR values between these two studies is precluded by the distinct descriptive statistics employed for data presentation (mean vs. median), it is noteworthy in the current investigation that the median ESR value is notably elevated. Then, based on the clinical condition and the type of laboratory alterations detected in the *Dirofilaria*/*Leishmania* active group, it is reasonable to suppose that the multisystemic effect of *L. infantum* in its active form may have had the greatest impact on the APR.

In the *Dirofilaria*/*Leishmania* group, the correlation between the ESR level and the other parameters, including markers of inflammation, differs greatly when considering the group as a whole or the subgroups based on the clinical form of CanL. In the *Dirofilaria*/*Leishmania* non-active group, ESR does not appear to correlate with any of the parameters. This is similar to the *Dirofilaria* group where the only correlation identified was with CRP. This finding may indicate the involvement of additional factors contributing to the observed increase in ESR within this group.

In the *Dirofilaria*/*Leishmania* active group, a strong and positive correlation between ESR and CRP, which is the main positive APP in dogs, as well as gamma globulins has been found. Increased concentrations of these serum proteins are known to occur in both CanL and HWD, being able to influence the ESR hastening agglutinations of erythrocyte as known from human medicine (38, 39). Additionally, a strong and negative correlation was discovered in the *Dirofilaria*/*Leishmania* active group between ESR and albumin, a negative APP whose reduction is expected during both CanL and HWD (7, 31, 40, 41). Albumin is thought to break up rouleaux and slow down red cell aggregation, resulting in a lower ESR and potentially explaining the correlation between clinical hypoalbuminemia and elevated ESR (42). However, this result varies from that obtained in heartworm-negative dogs affected by an active form of CanL, where no such correlation was found (21).

In the context of positive APP, a mention should be made of haptoglobin and ferritin. Although no statistically significant differences were found, it is noteworthy that in all groups herein considered, the number of animals with reduced haptoglobin was greater than those with an increased value (Table 4), despite being a positive APP. This paradoxical result is consistent with previous reports in heartworm-infected dogs (6, 10, 41), but further investigation is needed.

Ferritin has only been evaluated once in dogs with HWD, showing a moderate increase (41). Conversely, in dogs with leishmaniosis, an increase in ferritin is known (43, 44). In the canine population herein considered, no statistically significant differences were found, except for the number of animals with the increased parameter, which was significantly higher in the *Dirofilaria*/*Leishmania* active group. Considering that the precise mechanism underlying increases in serum ferritin is not well understood (45, 46) and the limited data available for specific diseases such as HWD, the role of ferritin as APP in heartworm-infected dogs as well as in *L. infantum* co-infected animals deserves additional research.

This study has a few limits, such as the small number of dogs included in the *Dirofilaria*/*Leishmania* active group. However, asymptomatic infection is the most common outcome of exposure to *Leishmania* parasites (47–49). Furthermore, the population enrolled in this study did not present overt clinical forms of HWD. Therefore, it is reasonable to suppose that the increase in ESR levels may be higher in heartworm-infected dogs with more severe clinical forms (e.g., pulmonary hypertension and caval syndrome), as has been demonstrated for CRP which increases according to the disease severity (11–13). Finally, the measurement of ESR in dogs has currently some limitations. Correction factors based on hematocrit values are lacking, and there is no information on the potential influence of key factors such as age, gender, and reproductive status of dogs on the ESR level. Additional research is required to elucidate these aspects which are known to affect the ESR, as reported in human medicine (50).

In conclusion, this study presents the first evaluation of ESR measured by a point-of-care device in dogs naturally infected by *D. immitis* and in a population of heartworm-infected and *L. infantum* seropositive animals. The ESR was confirmed to be an inflammation marker as well as a helpful disease index, being notably increased in heartworm-infected dogs affected by an active form of CanL, as previously reported (21). Further studies will be needed to assess the use of ESR in clinical settings, such as its potential as a follow-up tool for post-treatment monitoring.

## Data availability statement

The raw data supporting the conclusions of this article will be made available by the authors, without undue reservation.

## Ethics statement

The animal studies were approved by the Ethics Committee of the Department of Veterinary Medicine of the University of Bari (Prot. Uniba 24-2022). The studies were conducted in accordance with the local legislation and institutional requirements. Written informed consent was obtained from the owners for the participation of their animals in this study.

## Author contributions

MAC: Conceptualization, Data curation, Formal analysis, Funding acquisition, Project administration, Visualization, Writing – original draft. OG: Investigation, Data curation, Writing – review & editing. AU: Investigation, Writing – review & editing. FG: Writing – review & editing. VT: Investigation, Writing – review & editing. RD: Formal analysis, Writing – review & editing. MS: Writing – review & editing. AZ: Conceptualization, Funding acquisition, Project administration, Supervision, Writing – review & editing.
